# Intimate partner violence during pregnancy: prevalence and associated factors

**DOI:** 10.11606/s1518-8787.2020054002103

**Published:** 2020-10-28

**Authors:** Ranielle de Paula Silva, Franciéle Marabotti Costa Leite

**Affiliations:** I Universidade Federal do Espírito Santo Programa de Pós-Graduação em Saúde Coletiva VitóriaES Brasil Universidade Federal do Espírito Santo . Programa de Pós-Graduação em Saúde Coletiva . Vitória , ES , Brasil; II Universidade Federal do Espírito Santo Departamento de Enfermagem Programa de Pós-Graduação em Saúde Coletiva VitóriaES Brasil Universidade Federal do Espírito Santo . Departamento de Enfermagem . Programa de Pós-Graduação em Saúde Coletiva e Programa de Pós-graduação em Enfermagem. Vitória , ES , Brasil

**Keywords:** Pregnant women, Violence against women, Intimate partner violence, Domestic violence, Socioeconomic Factors

## Abstract

**OBJECTIVE:**

To identify the prevalence of violence during pregnancy and the association with the socioeconomic, behavioral and clinical characteristics of pregnant women.

**METHODS:**

Cross-sectional study in a low-risk maternity hospital in the municipality of Cariacica, Espírito Santo. A total of 330 puerperal women were interviewed from August to October 2017. Information on socioeconomic, behavioral, reproductive and clinical characteristics, as well as life experiences, was collected through a questionnaire. To identify the types of violence, the proper World Health Organization instrument was used. Gross bivariate and multivariate analysis was performed and adjusted for Poisson regression with robust variance.

**RESULTS:**

Prevalence was 16.1% (95%CI 2.5–20.4) for psychological violence, 7.6% (95%CI 5.1–11.0) for physical violence and 2.7% (95%CI 1.4–5.2) for sexual violence. Psychological violence remained associated with age, family income, beginning of sexual life, disease in pregnancy, desire to interrupt pregnancy and number of partners. Physical violence was associated with schooling, beginning of sexual life and disease in pregnancy. Sexual violence remained associated with marital status and desire to interrupt pregnancy (p < 0.05).

**CONCLUSIONS:**

Psychological violence by an intimate partner was the most prevalent among pregnant women. Women that were younger, had lower income and less schooling, who started their sexual life before the age of 14 and who wished to interrupt pregnancy, experienced violence more frequently during pregnancy.

## INTRODUCTION

Violence against women is recognized as one of the main forms of violation of human rights and may happen at any stage of life, including pregnancy ^1^ . The Pan American Health Organization ^2^ defines violence during pregnancy as aggression or threat of psychological, physical, or sexual abuse against pregnant women. Considered as a complex phenomenon and a public health problem, violence in this phase can negatively impact maternal and fetal health ^1 , 3^ .

It is worth considering that, among developed and developing countries, there is a variation in the prevalence of violence during pregnancy. In New Zealand, we can observe the prevalence of 15% ^4^ and7% in China ^5^ , while in Africa there is a prevalence of 2% to 57% ^6^ . In Brazil, the prevalence found varied from 13.1 to 34.6% ^7 , 8^ . A study conducted in 19 countries showed differences between high- and low-income countries, finding 2% in Australia and Denmark, but 8.1% and 13.5% in Colombia and Uganda, respectively ^9^ .

A direct relationship is observed between being a victim of violence and socioeconomic vulnerability and cultural factors ^10^: socioeconomic, sociodemographic and behavioral characteristics may increase the risk of being both perpetrator and victim of violence against women during pregnancy. Regarding women as victims, factors associated with young age, low education and income, low social support, unwanted pregnancy and history of family violence were considered ^7 , 11^ . Regarding the partner as an aggressor, observed associated factors were age, alcohol and illicit drug use ^11^ as well as unemployment.

It is important to highlight that the occurrence of violence during pregnancy affects women in a moment of physical and emotional fragility ^11^ , causing damage to their health. According to theliterature ^11 , 14^ , violence during pregnancy is associated with major obstetric problems, common mental disorder, postpartum depression and inadequate prenatal use. This damage also extends to the fetus, as it increases the risk of premature delivery and low birth weight ^15^ .

Therefore, considering the impact of violence on the health of both mother and child, and the lack of national publications on the problem of violence during pregnancy ^16^ , this study aimed to identify the prevalence of violence during pregnancy and verify the association with the socioeconomic, behavioral and clinical characteristics of pregnant women.

## METHODS

This is a cross-sectional study conducted in a low-risk maternity hospital in the municipality of Cariacica, Espírito Santo. The municipality is in the metropolitan region of the state, has a total area of approximately 280 km ^2^ , 348,738 inhabitants and human development index (HDI) of 0.718 ^17^ .

The sample consisted of puerperal women hospitalized with at least 24 hours after childbirth and live fetus (>500 grams) who had an intimate partner during pregnancy. An intimate partner is the partner or ex-partner, regardless of the formal bond, and boyfriends, if they maintained sexual relations.

The prevalence of intimate partner violence during pregnancy considered for the sample size was 20% ^16^ , confidence level of 95% and margin of error of 5%, plus 10% loss and 30% for confounding factors.

Data were collected from August to October 2017. The interviews took place privately and individually, and were conducted by female interviewers, previously trained to standardize the application of the questionnaire. The interviewers were supervised to ensure quality control and verify consistency in the completion of the questionnaires. At the end of each interview, the participants were given an informative folder about the types of violence and the main services for women in situations of violence.

Data were collected through a structured questionnaire, in which we asked about the following socioeconomic characteristics: age (14 to 19 years and 20 years or more), race (non-black and black), schooling level (up to 4 years and 5 years or more), marital status (without partner and with a partner), employment situation (no and yes) and monthly family income (less than a thousand reais and a thousand reais or more); behavioral characteristics regarding alcohol consumption during pregnancy (no and yes), smoking habit during pregnancy (no and yes) and history of illicit drug use (no and yes); reproductive and clinical aspects of the menarche (up to 13 years and 14 years or more), beginning of sexual life (up to 14 years and 15 years or more), number of pregnancies (1, 2, and 3 or more), history of sexually transmitted infections (STI) (no and yes), disease during pregnancy (no and yes), desire to interrupt the pregnancy (no and yes), number of partners in the latest year (1 and 2 or more); and the experience of sexual violence before the age of 15 (no and yes).

To investigate the violence experienced by women and perpetrated by an intimate partner, the World Health Organization instrument named Violence Against Women (WHO VAW) was applied, used to identify forms of violence in their psychological, physical and sexual domains. Through this instrument, the outcomes of the study were obtained: psychological, physical and sexual violence during pregnancy.

The data were analyzed using the Stata 13.0 statistical package. For the bivariate analyses, Pearson’s chi-square or Fisher’s exact test were used, according to any given assumption. In order to verify the association between psychological, physical and sexual violence during pregnancy and the independent variables, multivariate analysis was performed through Poisson regression with robust adjust of variance. The variables with p < 0.20 were included in the model and the permanence occurred when p < 0.05. The entry into the model occurred hierarchically, with the socioeconomic variables at the distal level, the reproductive and behavioral variables at the intermediate level and the experience of violence at the proximal level. The results were presented by gross prevalence ratio (PR) and adjusted with a 95% confidence interval (95%CI), an effect measure used for prevalence studies.

The study was approved by the Research Ethics Committee of the Cassiano Antônio de Moraes University Hospital, with opinion number 2,149,430.

## RESULTS

Psychological violence was the most prevalent, at 16.1% (95%CI 2.5–20.4), followed by physical violence at 7.6% (95%CI 5.1–11.0) and sexual violence at 2.7% (95%CI 1.4–5.2). Of the total sample studied, most women were 20 years or older (75.2%), declared themselves non-black (73.5%), had five years or more of schooling (73.0%) and lived with a partner (85.8%). About 77.0% were unemployed and 53.6% of the women had monthly family income equal to or greater than a thousand reais. Regarding behavioral aspects, most women did not drink or smoke during pregnancy and did not use illicit drugs in life (89.7%, 90.3% and 87.9%, respectively), as observed in Table1.

**Table 1:** Prevalence of psychological, physical and sexual violence against pregnant women, according to socioeconomic and behavioral characteristics. Cariacica, August to October 2017 (n = 330).

Characteristics of the woman			Psychological violence		Physical violence		Sexual violence	
Variables	N	%	% (95%CI)	p	% (95%CI)	p	% (95%CI)	p ^a^
Age (years)				0.002		0.068		0.031ª
14 to 19	82	24.8	26.8 (18.3–37.5)		12.2 (6.6–21.3)		6.1 (2.5–13.9)	
20 or more	248	75.2	12.5 (8.9–17.3)		6.1 (3.7–9.8)		1.6 (0.6–4.2)	
Race ^b^				0.937		0.109		0.473ª
Non-black	228	73.5	15.9 (9.4–25.5)		12.2 (6.6–21.3)		2.2 (0.9–5.2)	
Black	82	26.5	16.2 (12.0–21.6)		6.6 (4.0–10.7)		3.7 (1.2–10.8)	
Schooling level (years)				0.001		0.000		0.231ª
Up to 4 years	89	27.0	27.0 (18.7–37.2)		18.0 (11.3–27.5)		4.5 (1.7–11.5)	
5 or more	241	73.0	12.0 (8.5–16.8)		3.3 (1.9–7.0)		2.1 (0.9–4.9)	
Marital status				0.019		0.146		0.027ª
Without partner	47	14.2	27.7 (16.7–42.2)		12.8 (5.9–25.9)		8.5 (3.2–20.8)	
With a partner	283	85.8	14.1 (10.5–18.7)		6.7 (4.3–10.3)		1.8 (0.7–4.2)	
Employment situation				0.122		0.163		0.648ª
No	77	23.3	10.4 (5.2–19.5)		3.9 (1.2–11.5)		2.6 (0.6–9.9)	
Yes	253	76.7	17.8 (13.5–23.0)		8.7 (5.8–12.9)		2.8 (1.3–5.7)	
Monthly family income(reais)				0.001		0.557		0.216ª
< 1,000	153	46.4	23.5 (17.4–30.9)		8.5 (5.0–14.3)		3.9 (1.8–8.5)	
≥ 1,000	177	53.6	9.6 (6.0–14.9)		6.8 (3.9–11.6)		1.7 (0.5–5.2)	
Alcohol consumption during pregnancy				0.081		0.488ª		0.235ª
No	296	89.7	14.9 (11.2–19.4)		7.4 (4.9–11.1)		2.4 (1.1–4.9)	
Yes	34	10.3	26.5 (14.2–43.9)		8.8 (2.8–24.5)		5.9 (1.4–21.1)	
Smoking habit during pregnancy				0.003		0.070		0.395ª
No	298	90.3	14.1 (10.6–18.6)		6.7 (4.4–10.2)		3.0 (1.6–5.7)	
Yes	32	9.7	34.4 (19.9–52.4)		15.6 (6.5–32.9)		-	
History of illicit drug use				0.003		0.011		0.308ª
No	290	87.9	13.8 (10.3–18.3)		6.2 (3.9–9.7)		3.1 (1.6–5.9)	
Yes	40	12.1	32.5 (19.7–48.5)		17.5 (8.5–32.7)		-	

ª Fisher’s exact test

^b^ n = 310

95%CI: 95% confidence interval.

Table 2 shows that menarche before 13 years of age and the beginning of sexual life at 15 years or more occurred to about 73.0% of the participants. Approximately 38.0% had a previous pregnancy, most (83.0%) had no history of STI and no disease during pregnancy (77.6%). It was observed that 16.1% wished to interrupt pregnancy, 80.3% had a partner in the last year and91.5% did not suffer sexual violence before the age of 15.

**Table 2:** Prevalence of psychological, physical and sexual violence against women during pregnancy, according to clinical and reproductive characteristic and experience of violence. Cariacica, August to October 2017 (n = 330).

Characteristics of the woman			Psychological violence		Physical violence		Sexual violence	
Variables	N	%	% (95%CI)	p	% (95%CI)	p	% (95%CI)	p*
Menarche (years)				0,603		0,138*		0.245
Up to 13	240	72.7	15.4 (11.4–20.6)		8.8 (5.8–13.1)		3.3 (1.7–6.7)	
14 or more	90	27.3	17.8 (11.1–27.2)		4.4 (1.7–11.3)		1.1 (0.1–7.6)	
Beginning of sex life (years)				< 0.001		< 0.001		0.007
Up to 14	89	27.0	30.3 (21.6–40.7)		19.1 (12.2–28.7)		6.7 (3.0–14.3)	
15 or more	241	73.0	10.8 (7.4–15.4)		3.3 (1.7–6.5)		1.2 (0.4–3.8)	
Number of pregnancies				0.922		0.291		0.694
1	126	38.2	15.1 (9.8 - 22.5)		4.8 (2.1–10.3)		4.0 (1.7–9.2)	
2	94	28.5	17.0 (10.7–26.1)		8.5 (4.3–16.2)		2.1 (0.5–8.2)	
3 or more	110	33.3	16.4 (10.5–24.6)		10.0 (5.6–17.2)		1.8 (0.4–7.1)	
History of STI				< 0.001		0.126		0.183
No	274	83.0	12.0 (8.7–16.5)		6.6 (4.2–10.2)		2.2 (0.9–4.8)	
Yes	56	17.0	35.7 (24.2–49.1)		12.5 (6.0–24.2)		5.4 (1.7–15.5)	
Disease during pregnancy				0.001		0.028		0.327
No	256	77.6	12.5 (9.0–17.2)		5.9 (3.6–9.5)		2.3 (1.1–5.1)	
Yes	74	22.4	28.4 (19.2–39.8)		13.5 (7.4–23.5)		4.1 (1.3–12.0)	
Desire to interrupt the pregnancy				< 0.001		0.024		< 0.001
No	277	83.9	11.6 (8.3–15.9)		6.1 (3.8–9.7)		0.7 (0.1–2.9)	
Yes	53	16.1	39.6 (27.3–53.4)		15.1 (7.7–27.6)		13.2 (6.4–25.4)	
Number of sexual partners in the latest years				< 0.001		0.108		0.002
1	265	80.3	11.7 (8.3–16.2)		6.4 (4.0–10.1)		1.1 (0.4–3.5)	
2 or more	65	19.7	33.9 (23.3–46.2)		12.3 (6.2–22.9)		9.2 (4.2–19.2)	
Sexual violence before the age of 15				0.015		0.150*		0.172
No	302	91.5	14.6 (11.0–19.0)		7.0 (4.6–10.5)		2.3 (1.1–4.8)	
Yes	28	8.5	32.1 (17.4–51. 6)		14.3 (5.3–33.0)		7.1 (1.7–25.1)	

* Fisher’s exact test

95%CI: 95% confidence interval; STI: sexually transmitted infections.

Regarding the bivariate analysis, there was higher prevalence of psychological, physical and sexual violence in pregnant women who had the beginning of their sexual life before the age of 14 and who wished to interrupt pregnancy. Higher prevalence of psychological and physical violence during pregnancy occurred in those with less schooling, history of illicit drug use in life and who had disease during pregnancy. It is also possible to observe a higher frequency of psychological and sexual violence in pregnant adolescents, that have no partner, with a history of STI and history of sexual violence before 15 years of age. Regarding psychological violence alone, a higher frequency was observed in women with lower family income and who smoked during pregnancy. For sexual violence, a higher prevalence was observed among women who had two partners or more in the last year (p < 0.05). These data are presented in Tables 1 and 2.

After adjusting for the confounding variables, psychological violence remained associated with age, family income, beginning of sexual life, disease during pregnancy, desire to interrupt pregnancy and number of partners in the latest year. It was verified that psychological violence by an intimate partner during pregnancy was about twice as high (PR = 2.09; 95%CI 1.29–3.38) among pregnant adolescents (14 to 19 years) when compared with those aged 20 years or older. Regarding income, participants with monthly family income less than a thousand reais had 2.4 times more prevalence of psychological abuse than those with monthly income equal to or greater thana thousand reais. A prevalence of psychological violence was observed 87.0% higher in pregnant women who had the beginning of sexual life before the age of 14 years than in those who initiated at 15 years or more, 66.0% more frequent among those who had disease during pregnancy than among those who did not and twice as much (PR = 2.0; 95%CI 1.22–3.29) among those who wished to interrupt pregnancy compared to those who did not. Another association observed was with the number of sexual partners, with a higher frequency of psychological violence among women who had two or more partners in the latest year than among those who had one partner (PR = 1.82; 95%CI 1.10–3.00), as shown in Table 3 .

**Table 3:** Gross and adjusted analysis of the effects of socioeconomic, behavioral, reproductive and clinical variables, as well as experience, on psychological violence during pregnancy. Cariacica, August to October 2017 (n = 330).

Psychological violence				
Variables	Gross analysis		Adjusted analysis	
	Gross PR (95%CI)	p	Adjusted PR (95%CI)	p
Age (years)		0.002		0.007
14 to 19	2.14 (1.32–3.49)		2.09 (1.29–3.38)	
20 or more	1.0		1.0	
Schooling level (years)		0.001		0.066
Up to 4 years	2.24 (1.38–3.64)		1.63 (0.97–2.74)	
5 or more	1.0		1.0	
Marital status		0.016		0.084
Without partner	1.95 (1.13–3.38)		1.63 (0.94–2.84)	
With a partner	1.0		1.0	
Employment situation		0.137		0.882
No	1.71 (0.84–3.48)		0.97 (0.54–2.04)	
Yes	1.0		1.0	
Monthly family income(reais)		0.001		0.001
< 1,000	2.45 (1.43–4.18)		2.40 (1.41–4.08)	
≥ 1,000	1.0		1.0	
Alcohol consumption during pregnancy		0.070		0.816
No	1.0		1.0	
Yes	1.78 (0.95–3.32)		0.92 (0.47–1.81)	
Smoking habit during pregnancy		0.002		0.584
No	1.0		1.0	
Yes	2.44 (1.40–4.25)		1.19 (0.63–2.25)	
History of illicit drug use		0.002		0.187
No	1.0		1.0	
Yes	2.35 (1.38–4.01)		1.43 (0.84–2.45)	
Beginning of sex life (years)		< 0.001		0.011
Up to 14	2.81 (1.74–4.55)		1.87 (1.15–3.03)	
15 or more	1.0		1.0	
History of STI		< 0.001		0.413
No	1.0		1.0	
Yes	2.97 (1.84–4.77)		1.31 (0.69–2.50)	
Disease during pregnancy		0.001		0.049
No	1.0		1.0	
Yes	2.27 (1.40–3.69)		1.66 (1.01–2.75)	
Desire to interrupt the pregnancy		< 0.001		0.006
No	1.0		1.0	
Yes	3.43 (2.15–5.47)		2.0 (1.22–3.29)	
Number of sexual partners in the latest years		< 0.001		0.020
1	1.0		1.0	
2 or more	2.89 (1.80–4.65)		1.82 (1.10–3.00)	
Sexual violence before the age of 15		0.010		0.092
No	1.0		1.0	
Yes	2.21 (1.21–4.04)		1.66 (0.92–3.00)	

PR: prevalence ratio; 95%CI: 95% confidence interval; STI: sexually transmitted infections.

Regarding physical violence, schooling, beginning of sexual life and disease in pregnancy, these variables remained statistically associated after adjustment ( Table 4 ). It is noted that physical violence is about 4.5 times higher in pregnant women with up to four years of schooling than among those with five years or more (PR = 4.50; 95%CI 2.02–9.97). The prevalence of physical violence was 3.9 times higher in pregnant women with beginning of sexual life up to 14 years than in those who started at 15 years or older, and about twice as high in those who had disease during pregnancy than among those who did not (PR = 2.07; 95%CI 1.05–4.11).

**Table 4:** Gross and adjusted analysis of the effects of socioeconomic, behavioral, reproductive and clinical variables, as well as experience, on physical violence during pregnancy. Cariacica, August to October 2017 (n = 330).

Physical Violence				
Variables	Gross analysis		Adjusted analysis	
	Gross PR (95%CI)	p	Adjusted PR (95%CI)	p
Age (years)		0.071		0.237
14 to 19	2.02 (0.94–4.32)		1.58 (0.74–3.37)	
20 or more	1.0		1.0	
Race		0.112		0.209
Non-black	1.0		1.0	
Black	0.54 (0.25–1.15)		0.62 (0.29–1.31)	
Schooling level (years)		< 0.001		< 0.001
Up to 4 years	4.81 (2.20–10.51)		4.50 (2.02–9.97)	
5 or more	1.0		1.0	
Marital status		0.146		0.269
Without partner	1.90 (0.80–4.52)		1.58 (0.70–3.53)	
With a partner	1.0		1.0	
Employment situation		0.183		0.619
No	2.23 (0.69–7.27)		1.36 (0.41–4.55)	
Yes	1.0		1.0	
Smoking habit during pregnancy		0.069		0.416
No	1.0		1.0	
Yes	2.32 (0.94–5.79)		0.73 (0.34–1.57)	
History of illicit drug use		0.012		0.300
No	1.0		1.0	
Yes	2.82 (1.26–6.33)		1.65 (0.64–4.24)	
Menarche (years)		0.203		0.984
Up to 13	1.97 (0.69–5.59)		0.99 (0.36–2.74)	
14 or more	1.0		1.0	
Beginning of sex life (years)		< 0.001		0.002
Up to 14	5.75 (2.57–12.88)		3.90 (1.68–9.14)	
15 or more	1.0		1.0	
History of STI		0.127		0.281
No	1.0		1.0	
Yes	1.90 (0.83–4.34)		0.67 (0.32–1.39)	
Disease during pregnancy		0.031		0.037
No	1.0		1.0	
Yes	2.31 (1.08–4.92)		2.07 (1.05–4.11)	
Desire to interrupt the pregnancy		0.025		0.321
No	1,0		1,0	
Yes	2,46 (1,12–5,41)		1,60 (0,63–4,03)	
Number of sexual partners in the latest years		0,108		0,688
1	1,0		1,0	
2 or more	1,92 (0,86–4,25)		1,21 (0,47–3,12)	
Sexual violence before the age of 15		0,157		0,287
No	1,0		1,0	
Yes	2,05 (0,76–5,57)		1,48 (0,72–3,04)	

PR: prevalence ratio; 95%CI: 95% confidence interval; STI: sexually transmitted infections.

The occurrence of sexual violence was 3.8 times higher in those without a partner during pregnancy when compared to those with a partner (95%CI 1.06–13.40). Another finding was the prevalence of sexual violence about 15 times higher in women who wished to interrupt pregnancy than among those who did not ( Table 5 ).

**Table 5:** Gross and adjusted analysis of the effects of socioeconomic, behavioral, reproductive and clinical variables, as well as experience, on sexual violence during pregnancy. Cariacica, August to October 2017 (n = 330).

Sexual violence				
Variables	Gross analysis		Adjusted analysis	
	Gross PR (95%CI)	p	Adjusted PR (95%CI)	p
Age (years)				
14 to19	3.78 (1.04–13.77)	0.044	1.41 (0.81 – 10.62)	0.100
20 or more	1.0		1.0	
Marital status		0.016		0.041
Without partner	4.82 (1.34–17.32)		3.76 (1.06–13.40)	
With a partner	1.0		1.0	
Beginning of sex life (years)		0.015		0.127
Up to 14	5.42 (1.38–21.23)		3.17 (0.72–14.00)	
15 or more	1.0		1.0	
History of STI		0.197		0.238
No	1.0		1.0	
Yes	2.45 (0.63–9.51)		0.52 (0.18–1.54)	
Desire to interrupt the pregnancy		< 0.001		0.006
No	1.0		1.0	
Yes	18.29 (3.90–85.85)		14.90 (2.20–100.90)	
Number of sexual partners in the latest years		0.003		0.069
1	1.0		1.0	
2 or more	8.15 (2.09–31.80)		4.17 (0.90–19.43)	
Sexual violence before the age of 15		0.148		0.191
No	1.0		1.0	
Yes	3.08 (0.67–14.17)		2.20 (0.68–7.12)	

PR: prevalence ratio; 95%CI: 95% confidence interval; STI: sexually transmitted infections.

## DISCUSSION

During pregnancy, this study showed higher prevalence of psychological violence perpetrated by an intimate partner, followed by physical and sexual violence. The prevalence of psychological violence was 16.1%, a value like that found in Campinas (19.1%) ^10^ and Ribeirão Preto (14.7%) ^18^ . The frequency of physical violence was 7.4%, a percentage higher than that evidenced by a study conducted in a high-risk maternity hospital in Vitória (4.6%) ^19^ and like that found in Recife (7.4%) ^7^ . Sexual violence presented a lower frequency (2.7%); however, it was like the results presented in the literature ^20 , 21^ .

When analyzing the occurrence of violence according to age, it is noted that psychological violence was more frequent among adolescent women, corroborating the literature ^6 , 13^ . A study conducted in São Paulo observed that adolescent girls tend to naturalize violence in their relationships, as well as to perpetuate socially constructed gender norms, which makes them vulnerable to violence ^22^ . In addition, they are more vulnerable than older women due to economic dependence and restricted access to means of protection ^23^ . Similarly, women with lower monthly family income had a higher prevalence of psychological violence. Two systematic reviews found a direct relationship between lower socioeconomic status and violence by an intimate partner during pregnancy ^6 , 10^ . In this context, there is still a higher prevalence of physical violence among women with low schooling. This result corroborates what was found in another study, in which women with low schooling were twice as likely to suffer physical violence during pregnancy ^11^ .

Psychological and physical violence during pregnancy were associated with the beginning of sexual activity before the age of 14 years. Such findings are like those found in a study conducted by Durand and Schraiber ^24^ . Early beginning of sex life is considered a risk behavior for adolescents and may increase the chance of exposure to adverse situations throughout life ^25^ . It is also important to highlight that the occurrence of the first sexual act in the form of violence can generate damage to the emotional life of the victim, leading to behaviors of risk to their health and probable involvement with multiple partners at a young age ^24^ contributing to the increase of exposure to violence ^6^ .

Regarding disease during pregnancy, we identified that the puerperal women who went through it suffered psychological and physical violence by their partners more frequently. According to Audi et al. ^12^ , maternal morbidities related to obstetric problems, premature rupture of the membrane, urinary tract infection and vaginal bleeding were complications associated with violence during pregnancy. Physical injuries caused by violent acts of the partner are related to complications in pregnancy, such as placental detachment. In addition, stress during pregnancy can lead victims of violence to suffer from chronic and acute conditions. Violence can also interfere in an unhealthy lifestyle and inadequate eating habits, with negative consequences for the health of both mother and child ^7 , 10 , 12^ .

Having had two or more partners in the latest year increased the prevalence of psychological violence during pregnancy by 82%. From this perspective, a cohort study with pregnant women showed that having six or more intimate partners in life was associated with a higher occurrence of psychological violence ^13^ . A systematic review revealed a higher risk of violence for pregnant women with more than five intimate partners duringlife ^6^ .

In the present study, being a victim of psychological and sexual violence was associated with the desire to interrupt pregnancy. However, research shows that sexual violence during pregnancy is associated not only with the desire to interrupt it, but also with induced abortion. A study conducted in a public maternity hospital in Salvador with women who had abortions showed that 88% of them had suffered violence throughout their lives and 47% of them suffered some episode of violence during pregnancy ^26^ . In addition, data from a multicentric study revealed that women who had suffered sexual coercion throughout their lives had more abortions when compared to the group of women who had never suffered this type of violence ^27^ .

Unplanned pregnancy is also associated with mistreatment of pregnant women, since aggressiveness is much more related to the fact that the partner’s desire usually prevails regarding conception or abortion ^13^ . Disagreements between pregnant women and their partners regarding the acceptability of unplanned pregnancy, together with the impact of pregnancy on sexual life, can lead to verbal and emotional conflicts and, finally, to the occurrence of violence during pregnancy. In addition, women who suffer violence by an intimate partner may be more subject to forced or unprotected sex, incurring a greater number of unplanned pregnancies ^5^ .

Regarding marital status, sexual violence was observed more frequently among those who did not live with a partner. Similar data was found among Portuguese women ^28^ and a study conducted by Fiorotti et al. ^19^ showed that women without a partner had a 4.5 times higher prevalence of suffering physical violence during pregnancy. Another study observed that women without a partner had greater psychosocial problems during pregnancy than those with a partner. In addition, being married or in a stable union involves the existence of common values between the couple and a commitment to the formation of a family ^29^ .

As a limitation of the study, the cross-sectional nature stands out due to the causal relationship between exposure and outcome variables. It may also be possible to underestimate the prevalence due to information bias; however, the fact that the interview occurred in a private place contributes to the reduction of this bias. Another limiting factor refers to the fact that the study population was composed of women hospitalized in a low-risk public maternity, so the interpretation of the results should be done with caution.

Finally, it is worth noting the importance of this study when identifying the prevalence of violence committed by the partner during pregnancy and its associated factors, assisting in the elaboration of policies that might reduce and prevent the occurrence of this violence. Moreover, this research show that the violence practiced by a partner is present during pregnancy, and certain characteristics of women might make them more vulnerable to this phenomenon.

Based on this, health education actions are necessary to train health professionals in recognizing violence as a health problem and its impact on the health of the victim and the family. It is also essential to promote preventive actions and notification of violence against pregnant women to promote the disruption of this cycle. Thus, prenatal consultation can act as an essential tool in this process, as it provides opportunities for the detection of violence, and thus the possibility of comprehensive care. Another strategy that should be promoted is the broad and intersectoral debate on the theme of violence against women during pregnancy, using, for example, means of communication, contributing to the production of information in order to raise awareness and sensitize the population, as well as disseminate support and coping networks.
