# Supplementary material for: Intimate partner violence during pregnancy: prevalence and associated factors
Source: Rev Saude Publica. 2020 Oct 28;54:97. doi: 10.11606/s1518-8787.2020054002103 (PMC7593049; doi:10.11606/s1518-8787.2020054002103)
Supplement: Translation

# Translation: Violências por parceiro íntimo na gestação: prevalências e fatores associados

**Authors:** Ranielle de Paula Silva, Franciéle Marabotti Costa Leite

**Affiliations:** I Brasil Universidade Federal do Espírito Santo. Programa de Pós-Graduação em Saúde Coletiva. Vitória, ES, Brasil; II Brasil Universidade Federal do Espírito Santo. Departamento de Enfermagem. Programa de Pós-Graduação em Saúde Coletiva e Programa de Pós-Graduação em Enfermagem. Vitória, ES, Brasil

**Keywords:** Gestantes, Violência contra a Mulher, Violência por Parceiro Íntimo, Violência Doméstica, Fatores Socioeconômicos

## Abstract

**OBJETIVO:**

Identificar a prevalência das violências durante a gestação e verificar a associação com as características socioeconômicas, comportamentais e clínicas da gestante.

**MÉTODOS:**

Estudo transversal em uma maternidade de baixo risco do município de Cariacica, Espírito Santo. Foram entrevistadas 330 puérperas de agosto a outubro de 2017. Informações sobre as características socioeconômicas, comportamentais, reprodutivas e clínicas, assim como experiências de vida, foram coletadas por meio de questionário. Para identificar os tipos de violência, foi utilizado o instrumento da Organização Mundial da Saúde. Foi realizada análise bivariada e multivariada bruta e ajustada por regressão de Poisson com variância robusta.

**RESULTADOS:**

As prevalências foram 16,1% (IC95% 2,5–20,4) para violência psicológica, 7,6% (IC95% 5,1–11,0) para a física e 2,7% (IC95% 1,4–5,2) para a sexual. A violência psicológica manteve-se associada a idade, renda familiar, início da vida sexual, doença na gravidez, desejo de interromper a gestação e número de parceiros. A violência física esteve associada a escolaridade, início da vida sexual e doença na gravidez. Já a violência sexual manteve-se associada a situação conjugal e desejo de interromper a gestação (p < 0,05).

**CONCLUSÕES:**

A violência psicológica perpetrada pelo parceiro íntimo foi a de maior prevalência entre as gestantes. Mulheres mais jovens, com menor renda e escolaridade, que iniciaram a vida sexual até os 14 anos e que desejaram interromper a gravidez vivenciaram com maior frequência a violência durante a gestação.

## INTRODUÇÃO

A violência contra a mulher é reconhecida como uma das principais formas de violação dos direitos humanos e acomete qualquer fase da vida, inclusive a gestação^1^ . A Organização Pan-Americana da Saúde^2^ define violência na gravidez como agressão ou ameaça de abuso psicológico, físico ou sexual contra as mulheres grávidas. Considerada como fenômeno complexo e agravo de saúde pública, a violência nessa fase pode impactar negativamente na saúde materna e do feto^1 , 3^ .

Vale ponderar que, entre países desenvolvidos e em desenvolvimento, há uma variação na prevalência de violência durante a gestação. Observa-se na Nova Zelândia a prevalência de 15%^4^ e de 7% na China^5^ , enquanto na África verifica-se a prevalência de 2% a 57%^6^ . Já no Brasil, a prevalência encontrada é de 13,1 a 34,6%^7 , 8^ . Um estudo realizado em 19 países mostrou diferenças entre os países de alta e baixa renda, encontrando 2% na Austrália e Dinamarca, mas 8,1% e 13,5% na Colômbia e em Uganda, respectivamente^9^ .

Observa-se uma relação direta entre ser vítima de violência e vulnerabilidade socioeconômica e fatores culturais^10^: características socioeconômicas, sociodemográficas e comportamentais podem aumentar o risco de ser perpetrador e vítima de violência contra a mulher durante a gestação. No que tange à mulher como vítima, são considerados como fatores associados a idade jovem, baixa escolaridade e renda, baixo suporte social, gravidez indesejada e história de violência familiar^7 , 11^ . Já no que diz respeito ao parceiro como agressor, observam-se idade, uso de álcool e drogas ilícitas e não desempenhar trabalho remunerado^11^ como fatores associados.

Importante destacar que a ocorrência da violência no período gestacional acomete a mulher em um momento de fragilidade física e emocional^11^ , acarretando danos à sua saúde. De acordo com a literatura^11 , 14^ , a violência durante a gestação está associada a maiores problemas obstétricos, transtorno mental comum, depressão pós-parto e uso inadequado do pré-natal. Esse dano também se estende ao feto, pois aumenta o risco de parto prematuro e baixo peso ao nascer^15^ .

Diante disso, considerando o impacto da violência na gestação na saúde do binômio e ainda a carência de publicações nacionais sobre a problemática de violência na gravidez^16^ , o presente estudo teve como objetivo identificar a prevalência das violências durante a gestação e verificar a associação com as características socioeconômicas, comportamentais e clínicas da gestante.

## MÉTODOS

Trata-se de um estudo transversal realizado em uma maternidade de baixo risco no município de Cariacica, Espírito Santo. O município está localizado na região metropolitana do estado, possui área total de aproximadamente 280 km^2^, 348.738 habitantes e índice de desenvolvimento humano (IDH) de 0,718^17^ .

A amostra foi composta por puérperas internadas com no mínimo 24h de pós-parto e feto vivo (> 500 gramas) que tivessem tido parceiro íntimo durante a gestação. Considera-se como parceiro íntimo o companheiro ou ex-companheiro, independentemente do vínculo formal, e namorados, desde que mantendo relações sexuais.

Foram considerados para o cálculo do tamanho da amostra a prevalência de violência praticada pelo parceiro íntimo na gestação de 20%^16^ , nível de confiança de 95% margem de erro de 5%, acrescidos de 10% de perda e 30% para fatores de confusão.

A coleta de dados foi realizada no período de agosto a outubro de 2017. As entrevistas aconteceram individualmente e em local privativo por entrevistadoras do sexo feminino, previamente treinadas a fim de padronizar a aplicação do questionário. Houve a supervisão das entrevistadoras para assegurar o controle de qualidade e verificar a consistência no preenchimento dos questionários. Ao final de cada entrevista, era entregue às participantes um *folder* informativo sobre os tipos de violência e os principais serviços de atendimento às mulheres em situação de violência.

Os dados foram coletados por meio de questionário próprio estruturado, no qual se perguntava sobre as características socioeconômicas idade (14 a 19 anos e 20 anos ou mais), raça (não negra e negra), escolaridade (até 4 anos e 5 anos ou mais), situação conjugal (sem companheiro e com companheiro), trabalho remunerado (não e sim) e renda familiar mensal (menos de 1.000,00 reais e 1.000,00 reais ou mais); as características comportamentais consumo de álcool durante a gestação (não e sim), fumo durante a gestação (não e sim) e histórico de uso de droga ilícita (não e sim); os aspectos reprodutivos e clínicos menarca (até 13 anos e 14 anos ou mais), início da vida sexual (até 14 anos e 15 anos ou mais), número de gestações (1, 2, e 3 ou mais), histórico de infecção sexualmente transmissível (IST) (não e sim), doença na gravidez (não e sim), ter sentido desejo de interromper a gravidez (não e sim), número de parceiros no ano (1 e 2 ou mais); e a experiência de violência sexual antes dos 15 anos (não e sim).

Com o objetivo de investigar as violências vivenciadas pelas mulheres e perpetradas por parceiro íntimo, foi aplicado o instrumento da Organização Mundial da Saúde intitulado *World Health Organization Violence Against Women* (WHO VAW), usado para identificar as formas de violência em seus domínios psicológico, físico e sexual. Por meio desse instrumento, obtiveram-se os desfechos do estudo: violência psicológica, física e sexual durante a gestação.

Os dados foram analisados por meio do pacote estatístico Stata 13.0. Para as análises bivariadas, foram utilizados o teste de qui-quadrado de Pearson ou exato de Fisher, conforme o pressuposto. A fim de verificar a associação entre a violência psicológica, física e sexual durante a gestação e as variáveis independentes, foi realizada a análise multivariada por meio da regressão de Poisson com ajuste robusto da variância. As variáveis com p < 0,20 foram incluídas no modelo e a permanência se deu quando p < 0,05. A entrada no modelo aconteceu de forma hierárquica, estando no nível distal as variáveis socioeconômicas, no nível intermediário as variáveis reprodutivas e comportamentais e no nível mais proximal a experiência de violência. Os resultados foram apresentados por razão de prevalência (RP) bruta e ajustada com intervalo de confiança de 95% (IC95%), medida de efeito utilizada para estudos de prevalência.

O estudo foi aprovado pelo Comitê de Ética em Pesquisa do Hospital Universitário Cassiano Antônio de Moraes, com o parecer número 2.149.430.

## RESULTADOS

A violência psicológica foi a de maior prevalência, de 16,1% (IC95% 2,5–20,4), seguida da violência física com 7,6% (IC95% 5,1–11,0) e da violência sexual com 2,7% (IC95% 1,4–5,2). Do total da amostra estudada, a maioria das mulheres tinha 20 anos ou mais (75,2%), se declarou como não negra (73,5%), tinha cinco anos ou mais de estudo (73,0%) e residia com companheiro (85,8%). Cerca de 77,0% não tinham trabalho remunerado e 53,6% das mulheres tinham a renda familiar mensal igual ou maior que 1.000,00 reais. Quanto aos aspectos comportamentais, a maioria não consumiu álcool nem fumou durante a gestação e não fez uso de droga ilícita na vida (89,7%, 90,3% e 87,9%, respectivamente, conforme observado na Tabela 1 .

**Tabela 1:** Prevalência da violência psicológica, física e sexual contra a mulher na gestação, segundo características socioeconômicas e comportamentais. Cariacica, agosto a outubro de 2017 (n = 330).

Características da mulher			Violência psicológica		Violência física		Violência sexual	
Variáveis	n	%	% (IC95%)	p	% (IC95%)	p	% (IC95%)	p^a^
Idade (anos)				0,002		0,068		0,031
14 a 19	82	24,8	26,8 (18,3–37,5)		12,2 (6,6–21,3)		6,1 (2,5–13,9)	
20 ou mais	248	75,2	12,5 (8,9–17,3)		6,1 (3,7–9,8)		1,6 (0,6–4,2)	
Raça^b^				0,937		0,109		0,473
Não negra	228	73,5	15,9 (9,4–25,5)		12,2 (6,6–21,3)		2,2 (0,9–5,2)	
Negra	82	26,5	16,2 (12,0–21,6)		6,6 (4,0–10,7)		3,7 (1,2–10,8)	
Escolaridade (anos)				0,001		0,000		0,231
Até 4 anos	89	27,0	27,0 (18,7–37,2)		18,0 (11,3–27,5)		4,5 (1,7–11,5)	
5 ou mais	241	73,0	12,0 (8,5–16,8)		3,3 (1,9–7,0)		2,1 (0,9–4,9)	
Situação conjugal				0,019		0,146		0,027
Sem companheiro	47	14,2	27,7 (16,7–42,2)		12,8 (5,9–25,9)		8,5 (3,2–20,8)	
Com companheiro	283	85,8	14,1 (10,5–18,7)		6,7 (4,3–10,3)		1,8 (0,7–4,2)	
Trabalho remunerado				0,122		0,163		0,648
Não	77	23,3	10,4 (5,2–19,5)		3,9 (1,2–11,5)		2,6 (0,6–9,9)	
Sim	253	76,7	17,8 (13,5–23,0)		8,7 (5,8–12,9)		2,8 (1,3–5,7)	
Renda familiar mensal (reais)				0,001		0,557		0,216
< 1.000,00	153	46,4	23,5 (17,4–30,9)		8,5 (5,0–14,3)		3,9 (1,8–8,5)	
≥ 1.000,00	177	53,6	9,6 (6,0–14,9)		6,8 (3,9–11,6)		1,7 (0,5–5,2)	
Consumo de álcool durante a gestação				0,081		0,488^a^		0,235
Não	296	89,7	14,9 (11,2–19,4)		7,4 (4,9–11,1)		2,4 (1,1–4,9)	
Sim	34	10,3	26,5 (14,2–43,9)		8,8 (2,8–24,5)		5,9 (1,4–21,1)	
Fumo durante a gestação				0,003		0,070		0,395
Não	298	90,3	14,1 (10,6–18,6)		6,7 (4,4–10,2)		3,0 (1,6–5,7)	
Sim	32	9,7	34,4 (19,9–52,4)		15,6 (6,5–32,9)		-	
Histórico de uso droga ilícita				0,003		0,011		0,308
Não	290	87,9	13,8 (10,3–18,3)		6,2 (3,9–9,7)		3,1 (1,6–5,9)	
Sim	40	12,1	32,5 (19,7–48,5)		17,5 (8,5–32,7)		-	

^a^Teste exato de Fisher

^b^n = 310

IC95%: intervalo de confiança de 95%.

Na Tabela 2 , observa-se que a menarca antes dos 13 anos e o início da vida sexual aos 15 anos ou mais aconteceu para cerca de 73,0% das participantes. Aproximadamente 38,0% tiveram uma gestação, a maioria (83,0%) não tinha histórico de IST e não teve nenhuma doença na gravidez (77,6%). Observa-se que 16,1% desejaram interromper a gestação, 80,3% tiveram um parceiro no último ano e 91,5% não sofreram violência sexual antes dos 15 anos.

**Tabela 2:** Prevalência da violência psicológica, física e sexual contra a mulher na gestação, segundo características reprodutivas, clínicas e experiência de violência. Cariacica, agosto a outubro de 2017 (n = 330).

Características da mulher			Violência psicológica		Violência física		Violência sexual	
Variáveis	n	%	% (IC95%)	p	% (IC95%)	p	% (IC95%)	p*
Menarca (anos)				0,603		0,138*		0,245
Até 13	240	72,7	15,4 (11,4–20,6)		8,8 (5,8–13,1)		3,3 (1,7–6,7)	
14 ou mais	90	27,3	17,8 (11,1–27,2)		4,4 (1,7–11,3)		1,1 (0,1–7,6)	
Início da vida sexual (anos)				< 0,001		< 0,001		0,007
Até 14	89	27,0	30,3 (21,6–40,7)		19,1 (12,2–28,7)		6,7 (3,0–14,3)	
15 ou mais	241	73,0	10,8 (7,4–15,4)		3,3 (1,7–6,5)		1,2 (0,4–3,8)	
Número de gestações				0,922		0,291		0,694
1	126	38,2	15,1 (9,8 - 22,5)		4,8 (2,1–10,3)		4,0 (1,7–9,2)	
2	94	28,5	17,0 (10,7–26,1)		8,5 (4,3–16,2)		2,1 (0,5–8,2)	
3 ou mais	110	33,3	16,4 (10,5–24,6)		10,0 (5,6–17,2)		1,8 (0,4–7,1)	
Histórico de IST				< 0,001		0,126		0,183
Não	274	83,0	12,0 (8,7–16,5)		6,6 (4,2–10,2)		2,2 (0,9–4,8)	
Sim	56	17,0	35,7 (24,2–49,1)		12,5 (6,0–24,2)		5,4 (1,7–15,5)	
Doença na gravidez				0,001		0,028		0,327
Não	256	77,6	12,5 (9,0–17,2)		5,9 (3,6–9,5)		2,3 (1,1–5,1)	
Sim	74	22,4	28,4 (19,2–39,8)		13,5 (7,4–23,5)		4,1 (1,3–12,0)	
Desejou interromper a gestação				< 0,001		0,024		< 0,001
Não	277	83,9	11,6 (8,3–15,9)		6,1 (3,8–9,7)		0,7 (0,1–2,9)	
Sim	53	16,1	39,6 (27,3–53,4)		15,1 (7,7–27,6)		13,2 (6,4–25,4)	
Número de parceiros no ano				< 0,001		0,108		0,002
1	265	80,3	11,7 (8,3–16,2)		6,4 (4,0–10,1)		1,1 (0,4–3,5)	
2 ou mais	65	19,7	33,9 (23,3–46,2)		12,3 (6,2–22,9)		9,2 (4,2–19,2)	
Violência sexual antes dos 15 anos				0,015		0,150*		0,172
Não	302	91,5	14,6 (11,0–19,0)		7,0 (4,6–10,5)		2,3 (1,1–4,8)	
Sim	28	8,5	32,1 (17,4–51, 6)		14,3 (5,3–33,0)		7,1 (1,7–25,1)	

* Teste exato de Fisher

IC95%: intervalo de confiança de 95%; IST: infecções sexualmente transmissíveis.

Em relação à análise bivariada, observam-se maiores prevalências de violência psicológica, física e sexual em gestantes que tiveram o início da vida sexual antes dos 14 anos e que desejaram interromper a gravidez. Maiores prevalências de violência psicológica e física durante a gestação ocorreram naquelas com menor escolaridade, histórico de uso de droga ilícita na vida e que tiveram doença na gravidez. Também é possível observar maior frequência de violência psciológica e sexual em gestantes adolescentes, sem companheiro, com histórico de IST e violência sexual antes dos 15 anos de idade. No que tange somente à violência psicológica, foi observada maior frequência em mulheres com menor renda familiar e que fumaram durante a gestação. Já para a violência sexual, foi observada maior prevalência entre mulheres que tiveram dois parceiros ou mais no último ano (p < 0,05). Esses dados estão apresentados nas tabelas 1 e 2.

Nota-se, após o ajuste para as variáveis de confusão, que a violência psicológica se manteve associada à idade, renda familiar, início da vida sexual, doença na gravidez, desejo de interromper a gravidez e número de parceiros no último ano. Verifica-se que violência psicologia praticada pelo parceiro íntimo na gravidez foi cerca de duas vezes maior (RP = 2,09; IC95% 1,29–3,38) entre as gestantes adolescentes (14 a 19 anos) que entre aquelas com 20 anos ou mais. Quanto à renda, as participantes com renda familiar mensal menor que 1.000,00 reais tiveram 2,4 vezes mais prevalência de abuso psicológico que aquelas de renda mensal igual ou maior que 1.000,00 reais. Observa-se prevalência de violência psicológica 87,0% maior em gestantes que tiveram o início da vida sexual antes dos 14 anos do que naquelas que tiveram com 15 anos ou mais, 66,0% mais frequente entre as que tiveram doença na gravidez que entre as que não tiveram e duas vezes maior (RP = 2,0; IC95% 1,22–3,29) entre as que desejaram interromper a gravidez comparadas com as que não desejaram. Outra associação observada foi com o número de parceiros sexuais, constatando-se maior frequência da violência psicológica entre as mulheres que tiveram dois ou mais parceiros no último ano do que entre aquelas que tiveram um parceiro (RP = 1,82; IC95% 1,10–3,00), como apresentado na Tabela 3 .

**Tabela 3:** Análise bruta e ajustada dos efeitos das variáveis socioeconômicas, comportamentais, reprodutivas, clínicas e experiência sobre a violência psicológica durante a gestação. Cariacica, agosto a outubro de 2017.

Violência psicológica				
Variáveis	Análise bruta		Análise ajustada	
	RP bruta (IC95%)	p	RP ajustada (IC95%)	p
Idade (anos)		0,002		0,007
14 a 19	2,14 (1,32–3,49)		2,09 (1,29–3,38)	
20 ou mais	1,0		1,0	
Escolaridade (anos)		0,001		0,066
Até 4 anos	2,24 (1,38–3,64)		1,63 (0,97–2,74)	
5 ou mais	1,0		1,0	
Situação conjugal		0,016		0,084
Sem companheiro	1,95 (1,13–3,38)		1,63 (0,94–2,84)	
Com companheiro	1,0		1,0	
Trabalho remunerado		0,137		0,882
Não	1,71 (0,84–3,48)		0,97 (0,54–2,04)	
Sim	1,0		1,0	
Renda familiar mensal (reais)		0,001		0,001
< 1.000,00	2,45 (1,43–4,18)		2,40 (1,41–4,08)	
≥ 1.000,00	1,0		1,0	
Consumo de álcool durante a gestação		0,070		0,816
Não	1,0		1,0	
Sim	1,78 (0,95–3,32)		0,92 (0,47–1,81)	
Fumo durante a gestação		0,002		0,584
Não	1,0		1,0	
Sim	2,44 (1,40–4,25)		1,19 (0,63–2,25)	
Histórico de uso droga ilícita		0,002		0,187
Não	1,0		1,0	
Sim	2,35 (1,38–4,01)		1,43 (0,84–2,45)	
Início da vida sexual (anos)		< 0,001		0,011
Até 14	2,81 (1,74–4,55)		1,87 (1,15–3,03)	
15 ou mais	1,0		1,0	
Histórico de IST		< 0,001		0,413
Não	1,0		1,0	
Sim	2,97 (1,84–4,77)		1,31 (0,69–2,50)	
Doença na gravidez		0,001		0,049
Não	1,0		1,0	
Sim	2,27 (1,40–3,69)		1,66 (1,01–2,75)	
Desejou interromper a gestação		< 0,001		0,006
Não	1,0		1,0	
Sim	3,43 (2,15–5,47)		2,0 (1,22–3,29)	
Número de parceiros no ano		< 0,001		0,020
1	1,0		1,0	
2 ou mais	2,89 (1,80–4,65)		1,82 (1,10–3,00)	
Violência sexual antes dos 15 anos		0,010		0,092
Não	1,0		1,0	
Sim	2,21 (1,21–4,04)		1,66 (0,92–3,00)	

RP: razão de prevalências; IC95%: intervalo de confiança de 95%; IST: infecções sexualmente transmissíveis.

Quanto à violência física, a escolaridade, o início da vida sexual e doença na gravidez mantiveram-se estatisticamente associadas após o ajuste ( Tabela 4 ). Nota-se que a violência física é cerca de 4,5 vezes maior em gestantes com até quatro anos de estudo do que entre aquelas com cinco anos ou mais (RP = 4,50; IC95% 2,02–9,97). A prevalência de violência física foi 3,9 vezes maior em gestantes com o início da vida sexual até 14 anos do que naquelas que iniciaram com 15 anos ou mais, e cerca de duas vezes maior naquelas que tiveram doença na gravidez do que entre as que não tiveram (RP = 2,07; IC95% 1,05–4,11).

**Tabela 4:** Análise bruta e ajustada dos efeitos das variáveis socioeconômicas, comportamentais, reprodutivas, clínicas e experiência sobre a violência física durante a gestação. Cariacica, agosto a outubro de 2017.

Violência Física				
Variáveis	Análise bruta		Análise ajustada	
	RP bruta (IC95%)	p	RP ajustada (IC95%)	p
Idade (anos)		0,071		0,237
14 a19	2,02 (0,94–4,32)		1,58 (0,74–3,37)	
20 ou mais	1,0		1,0	
Raça		0,112		0,209
Não negra	1,0		1,0	
Negra	0,54 (0,25–1,15)		0,62 (0,29–1,31)	
Escolaridade (anos)		< 0,001		< 0,001
Até 4 anos	4,81 (2,20–10,51)		4,50 (2,02–9,97)	
5 ou mais	1,0		1,0	
Situação conjugal		0,146		0,269
Sem companheiro	1,90 (0,80–4,52)		1,58 (0,70–3,53)	
Com companheiro	1,0		1,0	
Trabalho remunerado		0,183		0,619
Não	2,23 (0,69–7,27)		1,36 (0,41–4,55)	
Sim	1,0		1,0	
Fumo durante a gestação		0,069		0,416
Não	1,0		1,0	
Sim	2,32 (0,94–5,79)		0,73 (0,34–1,57)	
Histórico de uso droga ilícita		0,012		0,300
Não	1,0		1,0	
Sim	2,82 (1,26–6,33)		1,65 (0,64–4,24)	
Menarca (anos)		0,203		0,984
Até 13	1,97 (0,69–5,59)		0,99 (0,36–2,74)	
14 ou mais	1,0		1,0	
Início da vida sexual (anos)		< 0,001		0,002
Até 14	5,75 (2,57–12,88)		3,90 (1,68–9,14)	
15 ou mais	1,0		1,0	
Histórico de IST		0,127		0,281
Não	1,0		1,0	
Sim	1,90 (0,83–4,34)		0,67 (0,32–1,39)	
Doença na gravidez		0,031		0,037
Não	1,0		1,0	
Sim	2,31 (1,08–4,92)		2,07 (1,05–4,11)	
Desejou interromper a gestação		0,025		0,321
Não	1,0		1,0	
Sim	2,46 (1,12–5,41)		1,60 (0,63–4,03)	
Número de parceiros no ano		0,108		0,688
1	1,0		1,0	
2 ou mais	1,92 (0,86–4,25)		1,21 (0,47–3,12)	
Violência sexual antes dos 15 anos		0,157		0,287
Não	1,0		1,0	
Sim	2,05 (0,76–5,57)		1,48 (0,72–3,04)	

RP: razão de prevalências; IC95%: intervalo de confiança de 95%; IST: infecções sexualmente transmissíveis.

A ocorrência de violência sexual foi 3,8 vezes maior naquelas sem companheiro durante a gestação quando comparadas às que tinham companheiro (IC95% 1,06–13,40). Outro achado foi a prevalência de violência sexual cerca de 15 vezes maior em mulheres que desejaram interromper a gestação do que entre as que não desejaram ( Tabela 5 ).

**Tabela 5:** Análise bruta e ajustada dos efeitos das variáveis socioeconômicas, comportamentais, reprodutivas, clínicas e experiência sobre a violência sexual durante a gestação. Cariacica, agosto a outubro de 2017.

Violência sexual				
Variáveis	Análise bruta		Análise ajustada	
	RP bruta (IC95%)	p	RP ajustada (IC95%)	p
Idade (anos)				
14 a 19	3,78 (1,04–13,77)	0,044	1,41 (0,81 – 10,62)	0,100
20 ou mais	1,0		1,0	
Situação conjugal		0,016		0,041
Sem companheiro	4,82 (1,34–17,32)		3,76 (1,06–13,40)	
Com companheiro	1,0		1,0	
Início da vida sexual (anos)		0,015		0,127
Até 14	5,42 (1,38–21,23)		3,17 (0,72–14,00)	
15 ou mais	1,0		1,0	
Histórico de IST		0,197		0,238
Não	1,0		1,0	
Sim	2,45 (0,63–9,51)		0,52 (0,18–1,54)	
Desejou interromper a gestação		< 0,001		0,006
Não	1,0		1,0	
Sim	18,29 (3,90–85,85)		14,90 (2,20–100,90)	
Número de parceiros no ano		0,003		0,069
1	1,0		1,0	
2 ou mais	8,15 (2,09–31,80)		4,17 (0,90–19,43)	
Violência sexual antes dos 15 anos		0,148		0,191
Não	1,0		1,0	
Sim	3,08 (0,67–14,17)		2,20 (0,68–7,12)	

RP: razão de prevalências; IC95%: intervalo de confiança de 95%; IST: infecções sexualmente transmissíveis.

## DISCUSSÃO

Durante a gestação foram evidenciadas na presente pesquisa maiores prevalências de violência psicológica perpetrada pelo parceiro íntimo, seguidas de violência física e sexual. A prevalência de violência psicológica encontrada foi de 16,1%, valor semelhante ao encontrado em Campinas (19,1%)^10^ e Ribeirão Preto (14,7%)^18^ . Já a frequência de violência física foi de 7,4%, percentual maior que o evidenciado por um estudo realizado em uma maternidade de alto risco em Vitória (4,6%)^19^ e semelhante ao encontrado em Recife (7,4%)^7^ . Quanto à violência sexual, esse tipo de agravo apresentou menor frequência (2,7%); todavia, foi similar aos resultados apresentados na literatura^20 , 21^ .

Ao analisar a ocorrência de violência de acordo com a idade, nota-se que a psicológica foi mais frequente entre as gestantes adolescentes, corroborando a literatura^6 , 13^ . Um estudo realizado em São Paulo observou que as adolescentes tendem a naturalizar a violência em suas relações, bem como a perpetuar as normas de gênero construídas socialmente, o que as torna vulneráveis à violência^22^ . Além disso, elas são mais vulneráveis que as mulheres mais velhas devido ao acesso restrito aos meios de proteção e à dependência econômica^23^ . Nesse mesmo sentido, no que tange à renda familiar, mulheres com menor renda familiar mensal tiveram maiores prevalência de violência psicológica. Duas revisões sistemáticas encontraram relação direta entre baixo status socioeconômico e violência por parceiro íntimo durante a gestação^6 , 10^ . Nesse contexto, ainda se observa a maior prevalência de violência física entre mulheres com baixa escolaridade. Esse resultado corrobora o encontrado em outro estudo, no qual as gestantes com baixa escolaridade apresentaram duas vezes mais chances de sofrer violência física na gestação^11^ .

A violência psicológica e física durante a gestação estiveram associadas ao ínicio da atividade sexual até os 14 anos, achados que se assemelham aos encontrados em um estudo realizado por Durand e Schraiber^24^ . O início precoce da relação sexual é considerado um comportamento de risco para os adolescentes, podendo aumentar a chance de exposição a situações adversas ao longo da vida^25^ . Também é importante destacar que a ocorrência do primeiro ato sexual na forma de violência pode gerar danos na vida emocional da vítima, provocando comportamentos de risco para sua saúde e, provavelmente, envolvimento com múltiplos parceiros em idade jovem^24^ , colaborando para o aumento da exposição à violência^6^ .

Com relação à doença na gravidez, identificamos que as puérperas que passaram por isso sofreram com maior frequência violência psicológica e física pelo parceiro. De acordo com Audi et al.^12^ , morbidades maternas relacionadas a agravos obstétricos, ruptura prematura de membrana, infecção do trato urinário e sangramento vaginal foram complicações associadas à ocorrência de violência na gestação. As lesões físicas causadas pelos atos violentos do parceiro estão relacionadas a complicações na gravidez, como descolamento de placenta. Além disso, o estresse no período gestacional pode levar as vítimas de violência a sofrerem de condições crônicas e agudas. Pode, ainda, interferir em um estilo de vida pouco saudável e em hábitos alimentares inadequados, com consequências negativas para a saúde do binômio mãe-filho^7 , 10 , 12^ .

Ter tido dois ou mais parceiros no último ano aumentou em 82% a prevalência de violência psicológica durante a gestação. Nessa perspectiva, um estudo de coorte com mulheres grávidas evidenciou que ter seis ou mais parceiros íntimos na vida esteve associado a maior ocorrência de violência psicológica^13^ . Uma revisão sistemática revelou um maior risco de violência para mulheres grávidas com mais de cinco parceiros íntimos durante a vida^6^ .

Na presente pesquisa, ser vítima de violência psicológica e sexual associou-se ao desejo de interromper a gestação. No entanto, pesquisas demonstram que sofrer violência sexual na gestação tem associação não só ao desejo de interrompê-la, mas também ao aborto provocado. Um estudo realizado em uma maternidade pública de Salvador com mulheres que provocaram aborto demonstrou que 88% delas haviam sofrido violência ao longo da vida e 47% delas sofreram algum episódio de violência durante a gestação^26^ . Além disso, dados de uma pesquisa multicêntrica revelou que mulheres que haviam sofrido algum tipo de coerção sexual ao longo da vida realizaram mais abortos quando comparadas ao grupo de mulheres que nunca sofreram^27^ .

A gravidez não planejada também está associada aos maus-tratos à gestante, uma vez que a agressividade está muito mais relacionada ao fato de o parceiro prevalecer quanto ao desejo ou não da gestação^13^ . Desentendimentos entre gestantes e seus parceiros quanto à aceitabilidade da gravidez não planejada, aliados ao impacto da gravidez na vida sexual, podem levar a conflitos verbais e emocionais e, finalmente, à ocorrência de violência durante a gravidez. Além disso, mulheres que sofrem violência por parceiros íntimos podem estar mais sujeitas ao sexo forçado ou desprotegido, incorrendo em um maior número de gestações não planejadas^5^ .

No que diz respeito à situação conjugal, a violência sexual foi observada com maior frequência entre aquelas que não residiam com companheiro. Achado semelhante foi encontrado entre portuguesas^28^ , e um estudo realizado por Fiorotti et al.^19^ mostrou que mulheres sem companheiro apresentaram prevalência 4,5 vezes maior de sofrer violência física na gestação. Outro estudo observou que mulheres sem companheiro tiveram maiores problemas psicossociais na gravidez do que aquelas com companheiro. Além disso, ser casada ou estar em uma união estável envolve a presença de valores em comum entre o casal e um compromisso com a formação de uma família^29^ .

Como limitação do estudo, destaca-se a natureza transversal por conta da relação de causalidade entre variáveis de exposição e desfecho. Também pode ser possível a subestimativa das prevalências devido ao viés de informação; contudo, a entrevista ter ocorrido em local privativo contribui para a redução desse viés. Outro fator limitante refere-se ao fato de a população do estudo ser composta por mulheres internadas em uma maternidade pública de baixo risco, por isso a interpretação dos resultados deve ser feita com cautela.

Por fim, vale salientar a importância deste estudo ao identificar as prevalências de violência cometida pelo parceiro durante a gestação e os fatores associados, auxiliando na elaboração de políticas a fim de reduzir e prevenir a ocorrência desse agravo. Além disso, os achados desta pesquisa evidenciam que a violência praticada pelo companheiro está presente na gestação, e determinadas características das mulheres podem torná-las mais vulneráveis a esse fenômeno.

A partir desses achados, são necessárias ações de educação em saúde, de modo a capacitar os profissionais de saúde no reconhecimento das violências como um problema de saúde e de seu impacto na saúde da vítima e na família. É também fundamental a promoção de ações preventivas e de notificação das violências contra a gestante a fim de promover o rompimento desse ciclo. Dessa forma, a consulta de pré-natal pode funcionar como ferramenta essencial nesse processo, pois oportuniza a detecção da violência, e assim, a possibilidade de cuidado integral. Outra estratégia que deve ser promovida é o debate de forma ampla e intersetorial da temática da violência contra a mulher durante a gestação, utilizando por exemplo os meios de comunicação, contribuindo para a produção de informações com o objetivo de conscientizar e sensibilizar a população, bem como divulgar as redes de apoio e enfrentamento.
